# Coronary Vasospasm in Eosinophilic Granulomatosis With Polyangiitis: A 4-Patient Case Series

**DOI:** 10.1016/j.jaccas.2026.110012

**Published:** 2026-08-28

**Authors:** Francesco Saverio Rea, Debora Russo, Concetta Mammone, Alfredo Fede, Mario Arieti, Michele Romano, Plinio Cirillo, Giovanni Esposito, Corrado Lettieri

**Affiliations:** a Department of Advanced Biomedical Sciences, University of Naples “Federico II”, Naples, Italy; b Division of Cardiology, Carlo Poma Hospital, Mantua, Italy

**Keywords:** acute coronary syndrome, coronary vasculitis, coronary vasospasm, eosinophilic granulomatosis with polyangiitis, hypereosinophilia

## Abstract

**Background:**

Coronary vasospasm is an uncommon manifestation of eosinophilic granulomatosis with polyangiitis (EGPA), and it may present as recurrent acute coronary syndrome (ACS).

**Case Summary:**

We report 4 patients presenting with recurrent ACS caused by angiographically documented coronary vasospasm. Three fulfilled the 2022 American College of Rheumatology/European Alliance of Associations for Rheumatology classification criteria for EGPA, whereas one was classified as having EGPA-like coronary inflammatory disease. Vasospastic episodes were refractory to conventional antianginal therapy but resolved rapidly after initiation of corticosteroids and immunosuppressive therapy. During a follow-up ranging from 2 to 6 years, no patient experienced recurrent cardiovascular events.

**Discussion:**

Recurrent ACS in patients with asthma, eosinophilia, or allergic disease should raise suspicion of eosinophilic coronary inflammatory disease. Early recognition and prompt immunosuppressive therapy may prevent recurrent myocardial injury and improve long-term outcomes.

**Take-Home Messages:**

Coronary vasospasm may represent the first manifestation of EGPA or EGPA-like coronary inflammatory disease. In patients with recurrent ACS and coexisting asthma or eosinophilic disease, clinicians should maintain a high index of suspicion for eosinophilic coronary inflammation, as timely immunosuppressive therapy is essential for sustained disease control.

## Visual Summary

**Figure:**
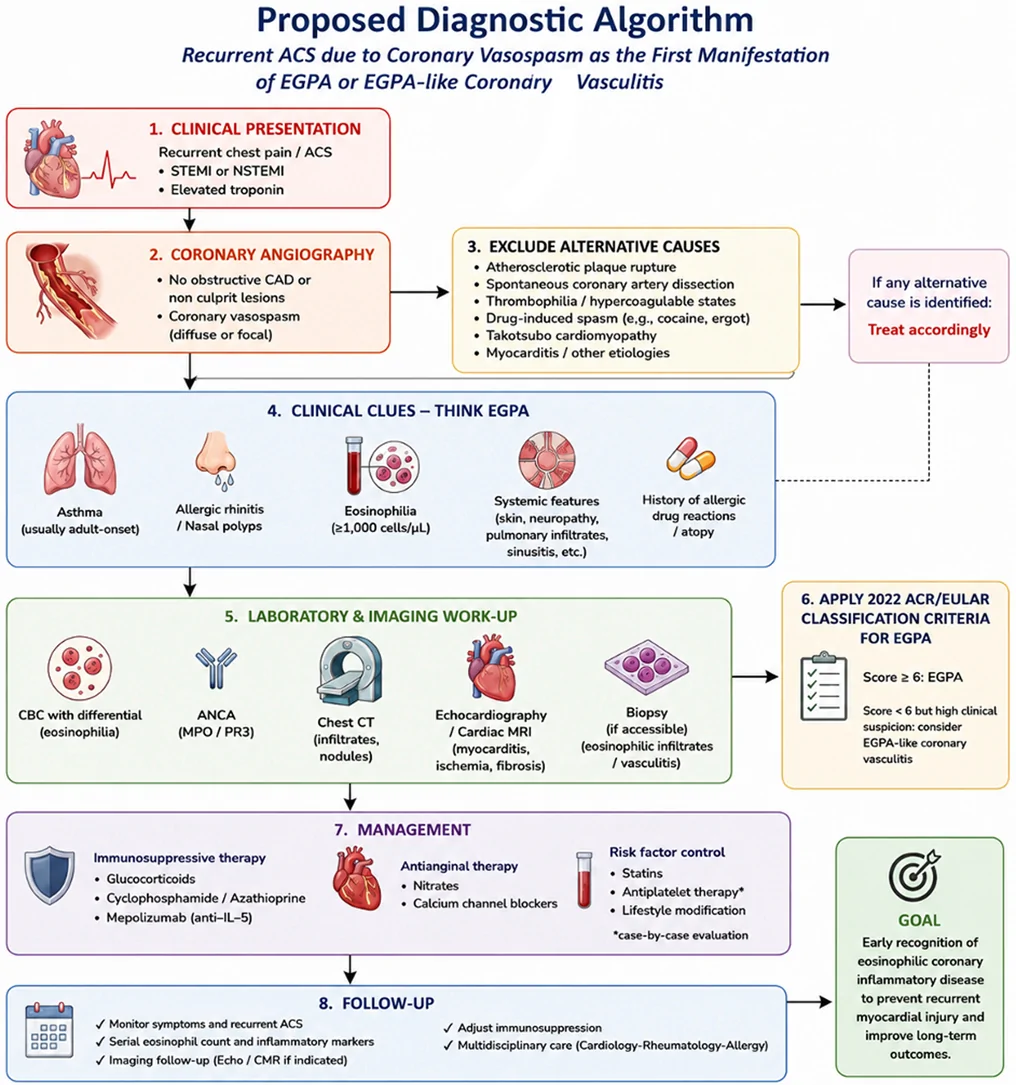

Eosinophilic granulomatosis with polyangiitis (EGPA) is a necrotizing vasculitis affecting small- and medium-sized vessels, characterized by asthma, eosinophilia, and eosinophilic tissue infiltration.^1^^,^^2^ Cardiac involvement is one of the strongest predictors of mortality. Although coronary involvement is uncommon, it may induce vasospasm, thrombosis, myocardial infarction, and recurrent acute coronary syndrome (ACS).^2^

Recurrent coronary vasospasm as the presenting manifestation of EGPA has been described almost exclusively in isolated case reports, and evidence from case series remains limited.

We retrospectively identified 4 consecutive patients presenting to our institution between 2018 and 2024 with recurrent ACS caused by angiographically documented coronary vasospasm and subsequently diagnosed with EGPA or EGPA-like coronary inflammatory disease (Table 1, Figure 1).

**Table 1:** Clinical Characteristics of 4 Patients

	Patient 1	Patient 2	Patient 3	Patient 4
Age, y	36	46	68	57
Sex	Male	Female	Female	Female
Previous hospital admission for ACS	Yes	Yes	Yes	Yes
Eosinophilia at index time	Yes	Yes	Yes	No
Nasal polyps	No	Yes	Yes	Yes
Asthma	Yes	Yes	Yes	Yes
First presentation	STEMI	STEMI	NSTEMI	Recurrent angina
Eosinophil count (cells/μL)	4,390	4,000	1,460	40
Pulmonary infiltrates	No	Yes	No	No
Coronary spasm at coronary angiography	Yes	Yes	Yes	Yes
Ejection fraction	51	63	60	60
Response to intracoronary nitrates	Yes	Yes	Yes	Yes
Response to immunosuppressive therapy	Yes	Yes	Yes	Yes

ACS = acute coronary syndrome; NSTEMI = non-ST-segment elevation myocardial infarction; STEMI = ST-segment elevation myocardial infarction.

**Figure 1:**
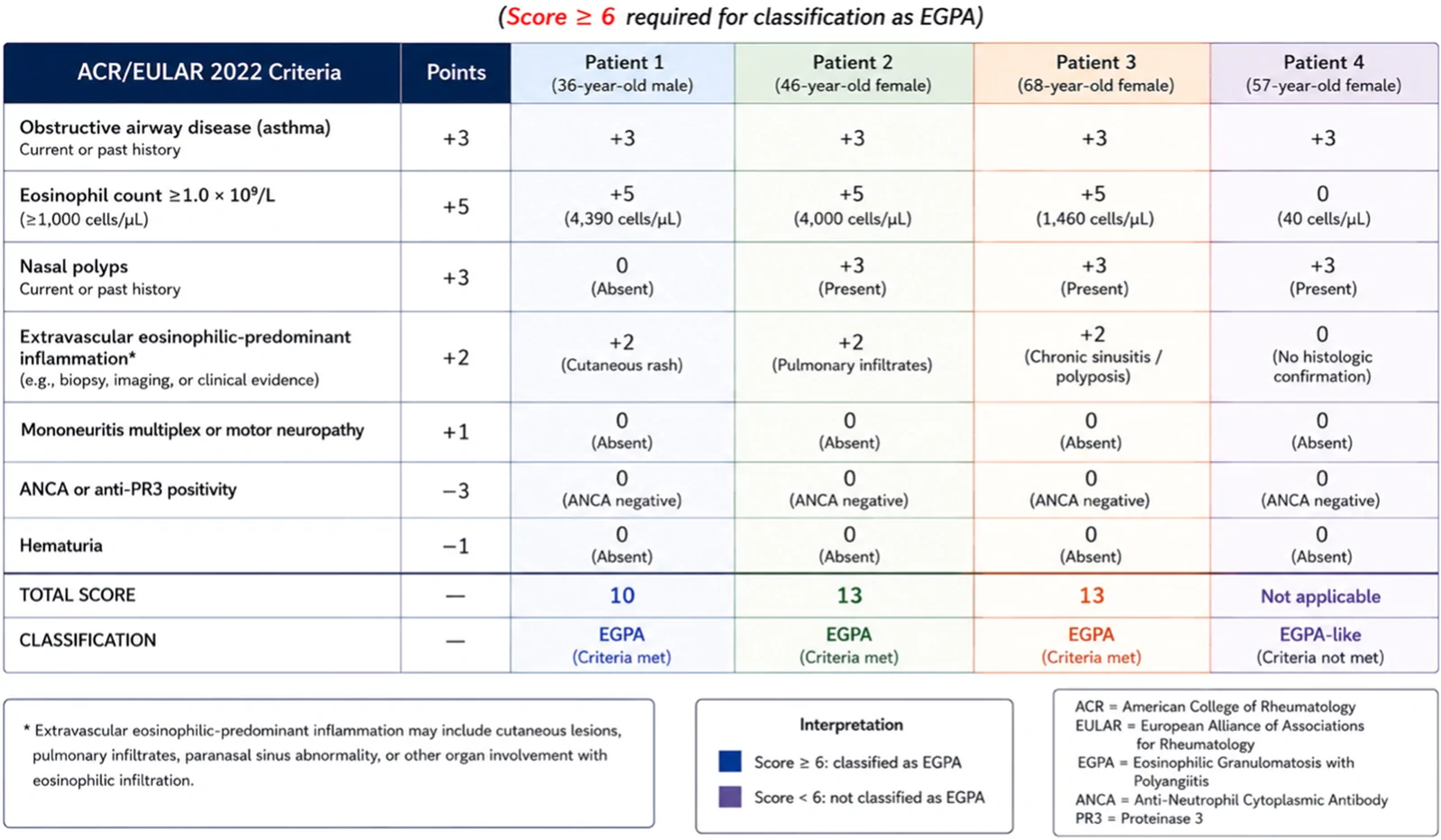
2022 ACR or EULAR Classification Criteria forEGPA in the 4 Patients The 2022 ACR/EULAR classification criteria were deemed inapplicable to patient 4 because a definite diagnosis of small- or medium-vessel vasculitis had not been established. Although the patient accrued 6 points from obstructive airway disease and nasal polyposis, she was categorized as having EGPA-like coronary inflammatory disease rather than definite EGPA. ANCA = antineutrophil cytoplasmic antibody; EGPA = eosinophilic granulomatosis with polyangiitis; PR3 = proteinase 3.

## Case 1

A 36-year-old Indian man with asthma treated with inhaled beclomethasone/formoterol, allergic rhinitis, Raynaud syndrome and a history of ketoprofen-induced skin rash presented with a 1-week history of chest pain. Electrocardiogram (ECG) showed sinus bradycardia without ischemic changes, while high-sensitivity troponin I was elevated (171 ng/L). He subsequently developed cardiogenic shock with anterolateral ST-segment elevation. Echocardiography revealed severe left ventricular dysfunction (ejection fraction: 20%). Emergency coronary angiography showed complete thrombotic occlusion of the left main coronary artery, successfully treated by primary percutaneous coronary intervention (PCI) with implantation of 2 drug-eluting stents. A mild aspirin-related skin reaction resolved with intravenous hydrocortisone. Left ventricular function rapidly recovered (ejection fraction: 51%), thrombophilia screening was negative, and the patient was discharged on dual antiplatelet therapy, atorvastatin, and bisoprolol.

Two weeks later, he was readmitted with recurrent nitrate-responsive chest and jaw pain. High-sensitivity troponin I was mildly elevated (106 ng/L), and repeat angiography showed patent stents without obstructive coronary disease. Recurrent ischemic episodes with transient ECG changes were associated with circumflex coronary vasospasm, which resolved completely after intracoronary nitroglycerin (Figure 2). Despite nitrates and calcium-channel blockers, vasospastic episodes persisted. Further investigations revealed marked eosinophilia (4,390 cells/μL), elevated C-reactive protein (9.4 mg/L), and positive antinuclear antibodies (ANA) (1:320). Antineutrophil cytoplasmic antibodies (ANCA) and the remaining autoimmune work-up were negative. Chest and abdominal computed tomography were unremarkable. Based on eosinophilia, asthma, and the 2022 American College of Rheumatology/European Alliance of Associations for Rheumatology (ACR/EULAR) criteria, EGPA was diagnosed. High-dose corticosteroid therapy promptly relieved symptoms. After sperm cryopreservation, intravenous cyclophosphamide (500 mg monthly for 6 months) was followed by maintenance therapy with azathioprine (100 mg/day).

**Figure 2:**
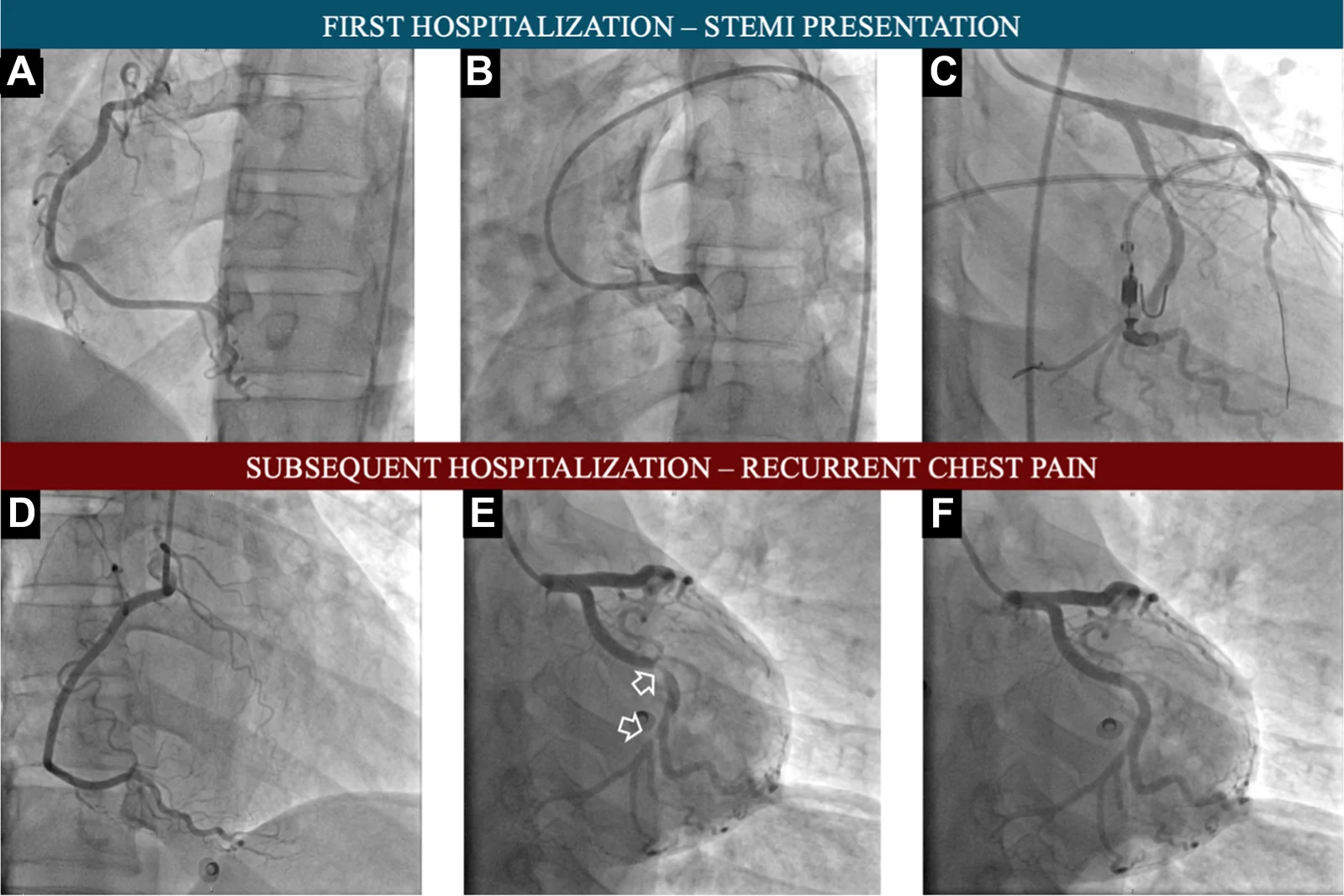
Patient 1: Coronary Angiography Findings at Initial and Subsequent Presentations First Event (A to C): Angiography performed during ST-segment elevation myocardial infarction (STEMI) presentation. (A) Right coronary artery without significant obstructive disease. (B) Acute thrombotic occlusion of the left main coronary artery. (C) Final angiographic result after primary percutaneous coronary intervention with implantation of 2 drug-eluting stents in the left main. Subsequent hospitalization (D to F): Angiography performed during recurrent chest pain in subsequent hospitalization. (D) Right coronary artery without significant obstructive disease. (E) Severe vasospasm of the circumflex coronary artery (white arrows). (F) Complete resolution of the circumflex vasospasm after intracoronary nitroglycerin administration.

At 6-year follow-up, the patient remained free of angina or dyspnea, with no recurrent hospitalizations.

## Case 2

A 46-year-old woman with a history of ischemic heart disease, bronchial asthma, nasal polyposis, transient pulmonary infiltrates, eosinophilia, and an adverse reaction to contrast medium presented to our outpatient clinic with recurrent chest pain and fluctuating high-sensitivity troponin I values documented on serial outpatient testing. Two years earlier, the patient had experienced a ST-segment elevation myocardial infarction (STEMI); coronary angiography performed at another hospital during that episode was interpreted as indicating a spontaneous dissection of the left anterior descending (LAD) artery and was treated with PCI and the implantation of 2 drug-eluting stents; however, in hindsight, we believe this event may have represented an earlier episode of coronary vasospasm rather than spontaneous coronary artery dissection. Considering the clinical presentation, medical history, and laboratory findings, the patient was admitted directly with a diagnosis of non-ST-segment elevation myocardial infarction (NSTEMI).

A repeat coronary angiography performed during this hospitalization revealed severe vasospasm at the geographical mismatch between the 2 stents in the mid-LAD, completely resolved with intracoronary nitroglycerin administration (Figure 3).

**Figure 3:**
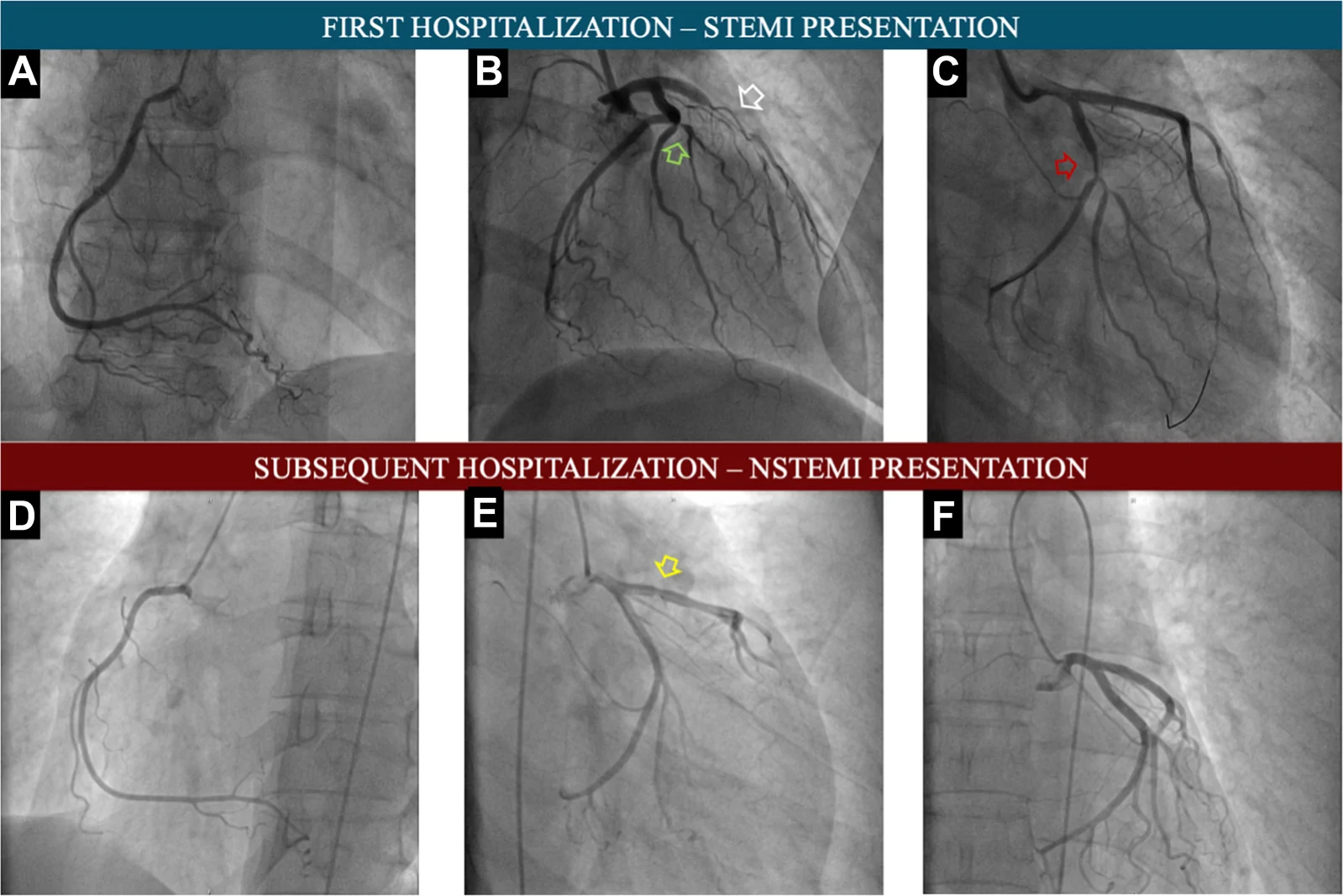
Patient 2: Coronary Angiography Findings at Initial And Recurrent Presentations First hospitalization (A to C): Angiography performed during ST-segment elevation myocardial infarction (STEMI) presentation. (A) Right coronary artery without significant obstructive disease. (B) Severe vasospasm of the left anterior descending (white arrow) and the circumflex (green arrow). (C) Final angiographic result after primary percutaneous coronary intervention with implantation of 2 drug-eluting stents in the left anterior descending and residual vasospasm of the circumflex (red arrow). Subsequent hospitalization (D to F): Angiography performed during non-ST-segment elevation myocardial infarction. (D) Right coronary artery without significant obstructive disease. (E) Left coronary artery with vasospasm in the geographical mismatch between the 2 drug-eluting stents in the mid left anterior descending (yellow arrow). (F) Complete resolution of left anterior descending vasospasm after intracoronary nitroglycerin administration.

Transthoracic echocardiography showed normal left ventricular wall thickness, preserved ejection fraction, normal global longitudinal strain, and no significant valvular abnormalities. During hospitalization, the patient developed a macular rash on the lower limbs. Laboratory testing revealed marked peripheral eosinophilia (4,000 cells/μL), positive antinuclear antibodies, and negative ANCA. Together with a history of asthma and nasal polyposis, these findings met the 2022 ACR/EULAR classification criteria for EGPA.

Treatment with corticosteroids (beclomethasone 100 mg/day), cyclophosphamide, transdermal nitroglycerin, and optimization of antianginal therapy led to rapid clinical improvement. Cyclophosphamide was discontinued and replaced with azathioprine, which was poorly tolerated because of gastrointestinal disturbances; therefore, mepolizumab 300 mg monthly was added to low-dose corticosteroid therapy (prednisone 12.5 mg/day).

At 3-year follow-up after the diagnosis of EGPA, the patient remained free of angina and dyspnea, with no recurrent hospitalizations.

## Case 3

A 68-year-old woman with a history of multiple drug allergies, autoimmune thyroiditis, erosive osteoarthritis, asthma, and vasomotor rhinitis requiring intermittent corticosteroids presented with NSTEMI.

Coronary angiography demonstrated severe mid LAD stenosis, which was treated with PCI and implantation of a drug-eluting stent. The patient was discharged 4 days later.

Two days after discharge, she developed urticarial wheals on the back and lower limbs. One week later, she was readmitted to our institution for recurrent NSTEMI. Repeat coronary angiography demonstrated diffuse vasospasm of the LAD, circumflex, and right coronary artery, with complete resolution after intracoronary nitroglycerin administration (Figure 4).

**Figure 4:**
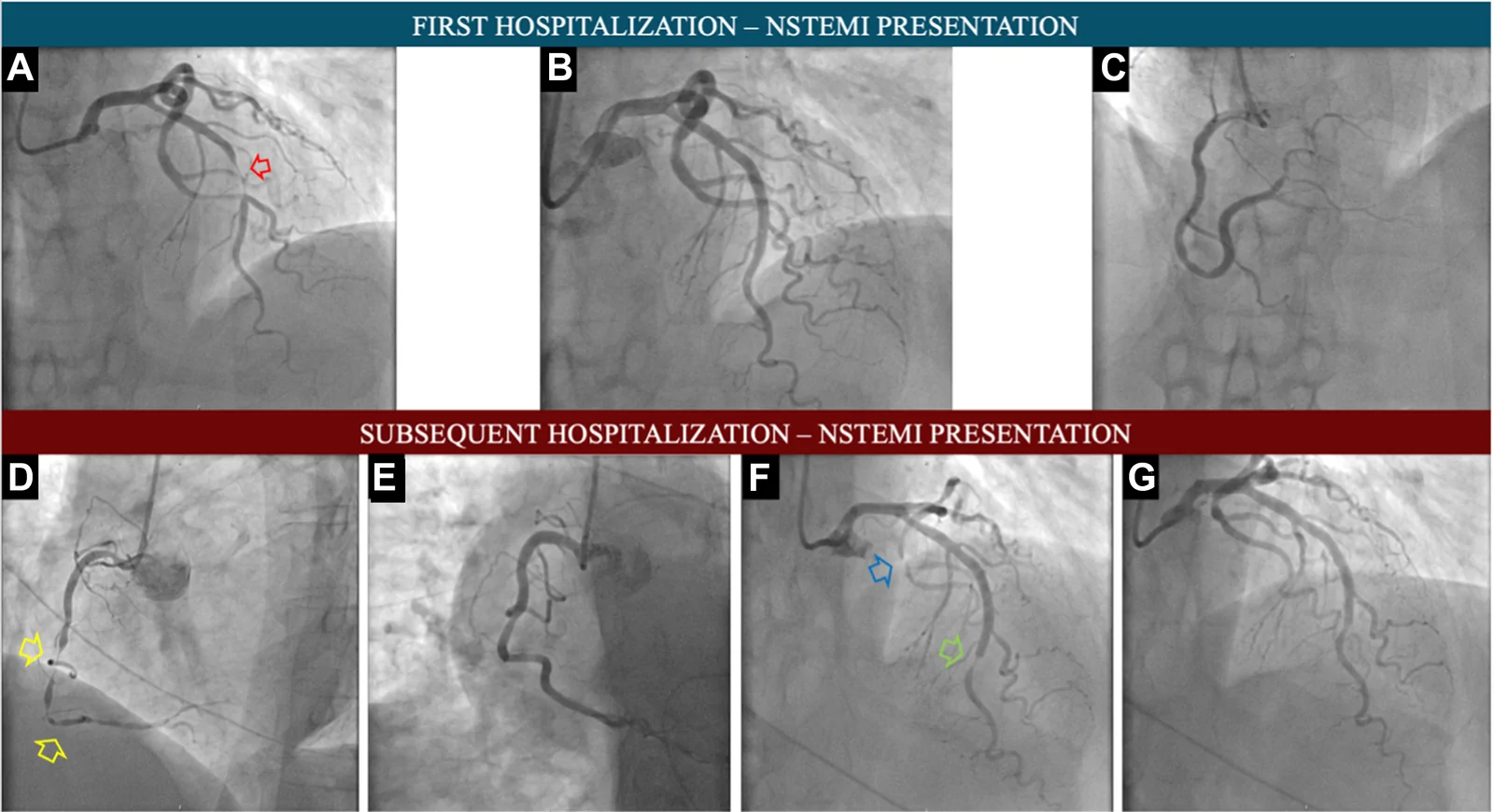
Patient 3: Coronary Angiography Findings at Initial And Recurrent Presentations First Hospitalization (A to C): Angiography performed during first non-ST-segment elevation myocardial infarction (NSTEMI) presentation. (A) Left coronary artery with severe stenosis of the left anterior descending (red arrow). (B) Final angiographic result after primary percutaneous coronary intervention with implantation of 2 drug-eluting stents in the left anterior descending. (C) Right coronary artery without significant obstructive disease. Subsequent hospitalization (D to G): Angiography performed during NSTEMI in subsequent hospitalization. (D) Right coronary artery with severe vasospasm (yellow arrows). (E) Complete resolution of right coronary artery vasospasm after intracoronary nitroglycerin administration. (F) Left coronary artery with severe vasospasm of the left anterior descending (green arrow) and the circumflex (blue arrow). (G) Complete resolution of left anterior descending and circumflex vasospasms after intracoronary nitroglycerin administration.

Transthoracic echocardiography showed a preserved left ventricular ejection fraction. Laboratory testing revealed peripheral eosinophilia. Allergologic and rheumatologic evaluations were performed, and computed tomography demonstrated paranasal sinus polyposis, which was subsequently confirmed by nasal endoscopy. Based on these findings, the patient met the 2022 ACR/EULAR classification criteria for EGPA.

Treatment with azathioprine 50 mg/day, mepolizumab 300 mg monthly, and corticosteroids (prednisone 12.5 mg/day) led to rapid and sustained clinical improvement.

At 3-year follow-up after the diagnosis of EGPA, the patient remained free of angina and dyspnea, with no recurrent hospitalizations.

## Case 4

A 57-year-old woman with severe corticosteroid-dependent asthma, nasal polyposis, aspirin intolerance, treated epilepsy, and a family history of cardiovascular disease presented with recurrent angina. Initial coronary angiography showed normal coronary arteries, and computed tomography excluded aortic dissection and pulmonary embolism.

Two months later, she developed an inferior STEMI. Repeat angiography showed no obstructive coronary artery disease. An extensive work-up for systemic vasculitis, including autoimmune serology and nasal polyp histology, was inconclusive. She was discharged on clopidogrel, statin/ezetimibe, isosorbide mononitrate, diltiazem, prednisone, and budesonide/formoterol.

Four months later, she was readmitted with recurrent chest pain despite antianginal therapy. ECG showed transient ST-segment depression in leads V_5_ and V_6_, and high-sensitivity troponin I peaked at 924 ng/L, consistent with NSTEMI. Angiography during symptoms demonstrated diffuse mid-right coronary artery vasospasm, which resolved completely after intracoronary and sublingual nitrates (Figure 5).

**Figure 5:**
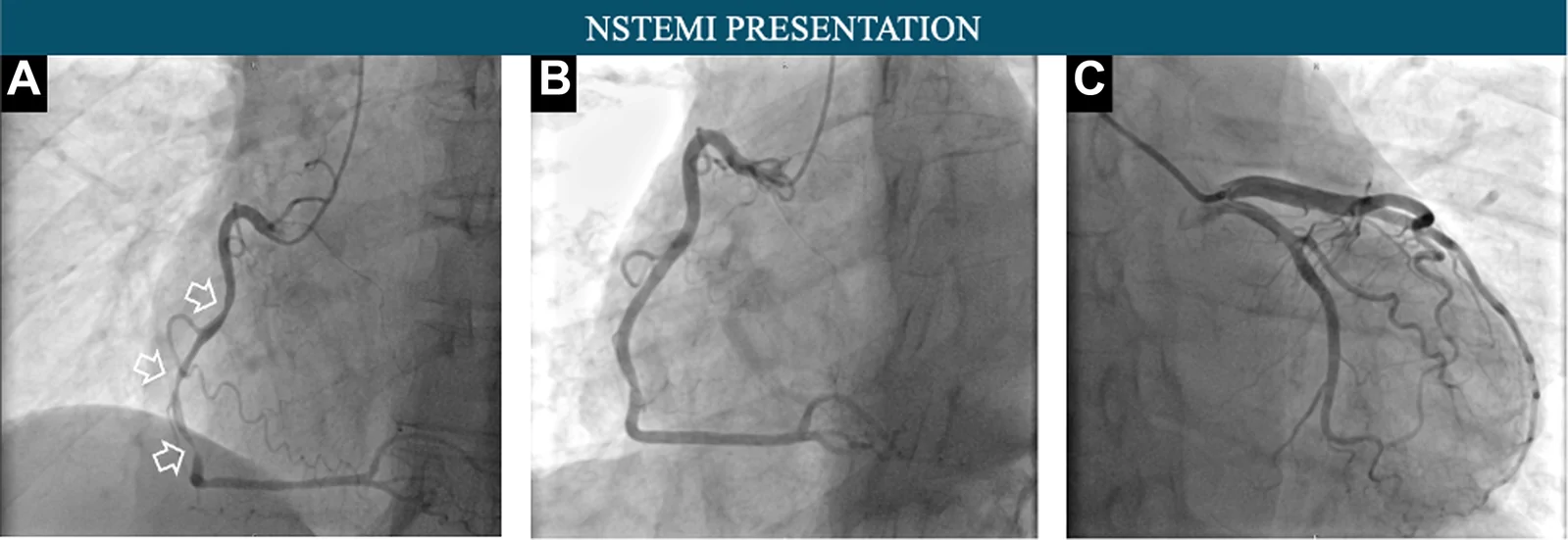
Patient 4: Coronary Angiography Findings During NSTEMI Presentation (A) Right coronary artery with diffuse, severe vasospasm (white arrows). (B) Complete resolution of right coronary artery vasospasm after intracoronary nitroglycerin administration. (C) Left coronary artery without significant obstructive disease. NSTEMI = non-ST-segment elevation myocardial infarction.

Echocardiography and cardiac magnetic resonance showed mild inferior basal hypokinesia with preserved left ventricular systolic function. Although peripheral eosinophilia, ANCA positivity, and histological evidence of vasculitis were absent, the combination of severe asthma, nasal polyposis, recurrent angiographically documented vasospasm without obstructive coronary disease, and sustained response to prednisone (12.5 mg/day) supported an EGPA-spectrum inflammatory coronary disorder. As the 2022 ACR/EULAR criteria were not formally met, multidisciplinary evaluation led to a diagnosis of EGPA-like coronary inflammatory disease. Corticosteroid therapy was continued, and the patient remained event-free at 2-year follow-up.

## Discussion

This 4-patients case series highlights coronary vasospasm as a presenting manifestation of EGPA and EGPA-like coronary inflammatory disease, emphasizing a rare but clinically important phenotype that requires prompt recognition and targeted immunosuppressive therapy. Although cardiac involvement in EGPA is a well-established predictor of mortality, isolated coronary vasculitis causing recurrent ACS remains underrecognized, and the available evidence is largely limited to isolated case reports.^1^, ^2^, ^3^

### Pathophysiological mechanisms

The mechanisms underlying coronary vasospasm in eosinophilic disorders are multifactorial and differ substantially from those of atherosclerotic coronary disease. Eosinophils infiltrate the coronary arterial wall and release cytotoxic granule proteins, causing direct endothelial injury.^2^ This inflammatory process promotes endothelial dysfunction by reducing nitric oxide bioavailability, releasing vasoactive mediators such as leukotrienes, and activating complement, ultimately resulting in coronary vasospasm.^3^ The predominance of inflammation in the adventitia may explain why coronary angiography is frequently normal or shows only dynamic abnormalities rather than fixed stenoses.^4^ Furthermore, eosinophil degranulation promotes a prothrombotic milieu, predisposing to mural thrombosis and, in selected cases, spontaneous coronary artery dissection.^2^

### Clinical phenotype and diagnostic considerations

The clinical features in this series distinguish eosinophilic coronary vasculitis from typical vasospastic angina (Figure 6). All patients had asthma and allergic manifestations, consistent with the recognized association between cardiac involvement and ANCA-negative, eosinophil-predominant EGPA.^5^^,^^6^ Three patients also had marked peripheral eosinophilia, supporting prior observations that higher eosinophil counts correlate with cardiac involvement.^1^^,^^7^ Vasospastic episodes were refractory to conventional vasodilator therapy and recurred despite optimization of antianginal treatment, a pattern that should raise suspicion for an underlying inflammatory process rather than primary vasospastic angina.^2^^,^^3^ Notably, recurrence occurred early after the initial presentation, often within days or weeks, suggesting ongoing active vascular inflammation.

**Figure 6:**
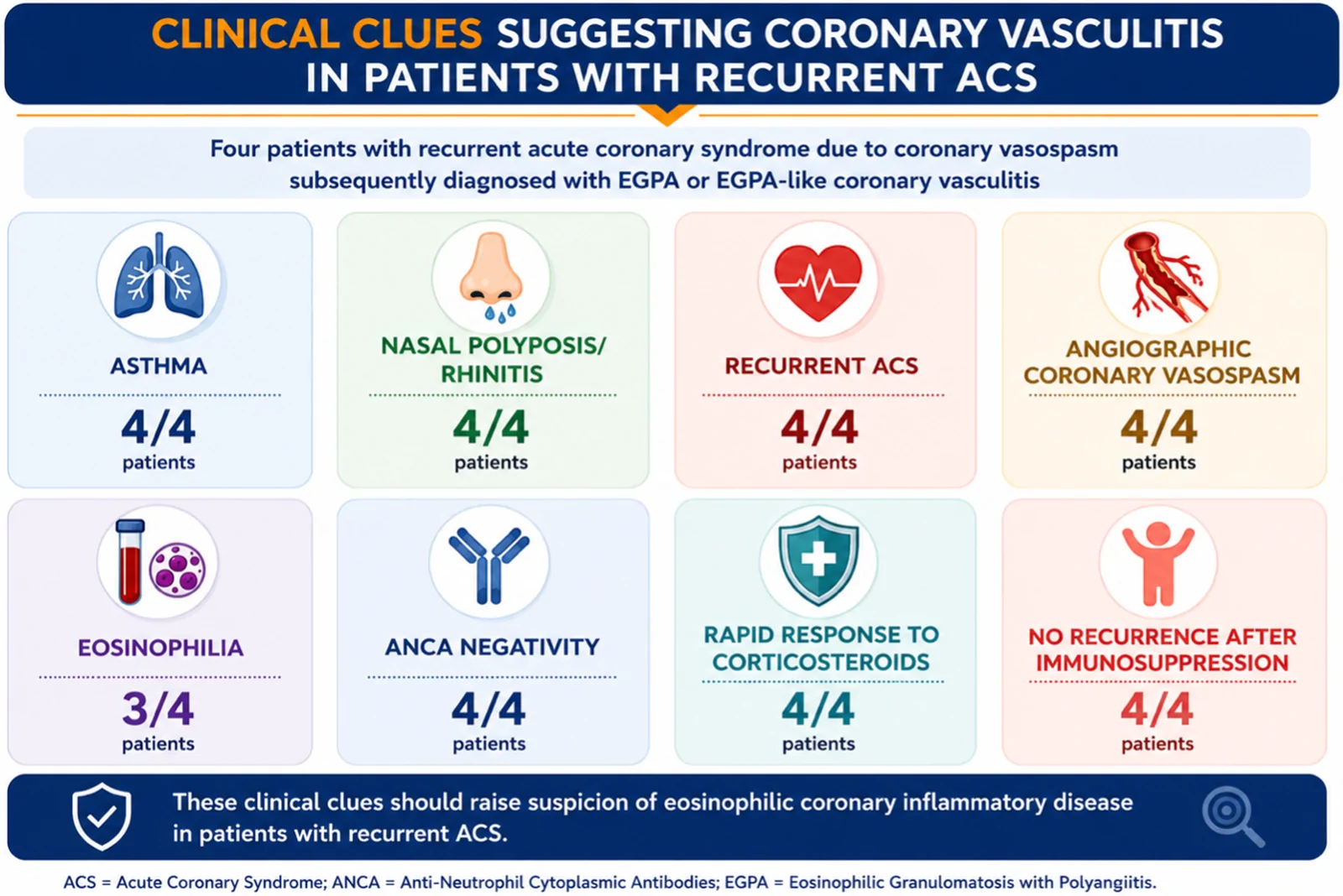
Clinical Clues Suggesting Coronary Vasculitis In Patients With Recurrent ACS ACS = acute coronary syndrome; ANCA = antineutrophil cytoplasmic antibody; EGPA = eosinophilic granulomatosis with polyangiitis.

Three patients fulfilled the 2022 ACR/EULAR criteria for EGPA. The fourth was classified as EGPA-like coronary inflammatory disease because classification criteria were not formally met despite a compatible phenotype and sustained response to corticosteroids. This distinction also highlights that classification criteria are primarily intended for research purposes and should not be used in isolation to establish a clinical diagnosis. Another notable finding was the absence of ANCA positivity in all 4 patients, consistent with previous reports that cardiac involvement occurs predominantly in ANCA-negative EGPA.^1^^,^^5^^,^^6^ This inverse association likely reflects distinct pathogenic pathways: ANCA-positive disease is mainly characterized by necrotizing vasculitis involving the kidneys and peripheral nervous system, whereas ANCA-negative disease is driven predominantly by eosinophilic tissue infiltration and is more frequently associated with cardiac involvement.^6^

Alternative diagnoses, including primary vasospastic angina, Kounis syndrome, drug- or contrast-induced vasospasm, post-PCI stent-edge spasm, spontaneous coronary artery dissection, thrombophilia, hypereosinophilic syndrome, and myocarditis, were systematically considered according to each patient's clinical presentation. These diagnoses were considered unlikely based on the absence of consistent triggering factors, negative ancillary investigations when appropriate, the presence of asthma and eosinophilic disease, angiographically documented recurrent vasospasm, and the prompt, sustained response to immunosuppressive therapy. Although eosinophilic myocarditis was considered in the differential diagnosis, imaging findings, including preserved ventricular function in most patients and the absence of CMR features suggestive of active myocarditis when performed, together with angiographically documented vasospasm during ischemic symptoms, supported myocardial injury secondary to coronary vasospasm rather than primary myocardial inflammation.

### Therapeutic implications and outcomes

The most clinically relevant finding in this series is the marked response to immunosuppressive therapy and the limited efficacy of conventional antianginal treatment alone. All patients experienced complete resolution of symptoms after initiating corticosteroids, often within days, and remained free of recurrent cardiovascular events throughout long-term follow-up (2-6 years) while receiving maintenance immunosuppressive therapy.

Initial treatment relies on high-dose corticosteroids to rapidly suppress eosinophilic vascular inflammation and prevent further myocardial injury.^8^^,^^9^ For patients with severe or refractory disease, additional immunosuppressive therapy is recommended. In our series, cyclophosphamide was used for induction therapy, followed by maintenance with azathioprine or mepolizumab according to disease severity in patients with cardiac involvement.^10^ Conventional antianginal medications remain important adjunctive therapies but are insufficient when used without immunosuppression.

Historically, cardiac involvement in EGPA was associated with high mortality, accounting for approximately half of EGPA-related deaths.^9^ More recent studies, however, have shown substantially improved outcomes with earlier diagnosis and contemporary immunosuppressive strategies. In our series, all patients remained asymptomatic throughout follow-up, with no recurrent cardiovascular events or hospitalizations. These favorable outcomes likely reflect early recognition, prompt initiation of immunosuppressive therapy, and careful multidisciplinary management. Nevertheless, cardiac involvement remains a marker of severe disease and warrants cautious long-term surveillance, as major adverse cardiovascular events continue to occur in some patients despite treatment.

### Clinical implications

Coronary vasospasm may be the initial manifestation of EGPA or EGPA-like coronary inflammatory disease, preceding the onset of overt systemic disease. Second, recurrent ACS despite optimal antianginal therapy in patients with asthma, allergic manifestations, or eosinophilia should prompt consideration of eosinophilic coronary inflammatory disease. Third, conventional vasodilator therapy alone is insufficient for disease control, whereas timely immunosuppressive treatment appears essential to prevent recurrent myocardial injury. Finally, multidisciplinary collaboration among interventional cardiologists, rheumatologists, and allergists/immunologists proved essential for establishing the diagnosis, selecting immunosuppressive therapy, and coordinating long-term follow-up, highlighting the value of an integrated approach in these complex patients.

A further limitation of this case series is the lack of intracoronary imaging (intravascular ultrasound/optical coherence tomography), which might have provided additional mechanistic insights and helped exclude alternative causes of ACS in selected cases.

## Conclusions

Coronary vasospasm causing ACS may be the initial manifestation of EGPA and EGPA-like coronary inflammatory disease. Recurrent ACS despite optimal antianginal therapy in patients with specific clinical characteristics should raise suspicion of an eosinophilic coronary inflammatory disease. Early recognition and timely initiation of immunosuppressive therapy may prevent recurrent myocardial injury and improve long-term outcomes. A multidisciplinary team is essential for establishing the diagnosis and optimizing patient management.

**Visual Summary:**
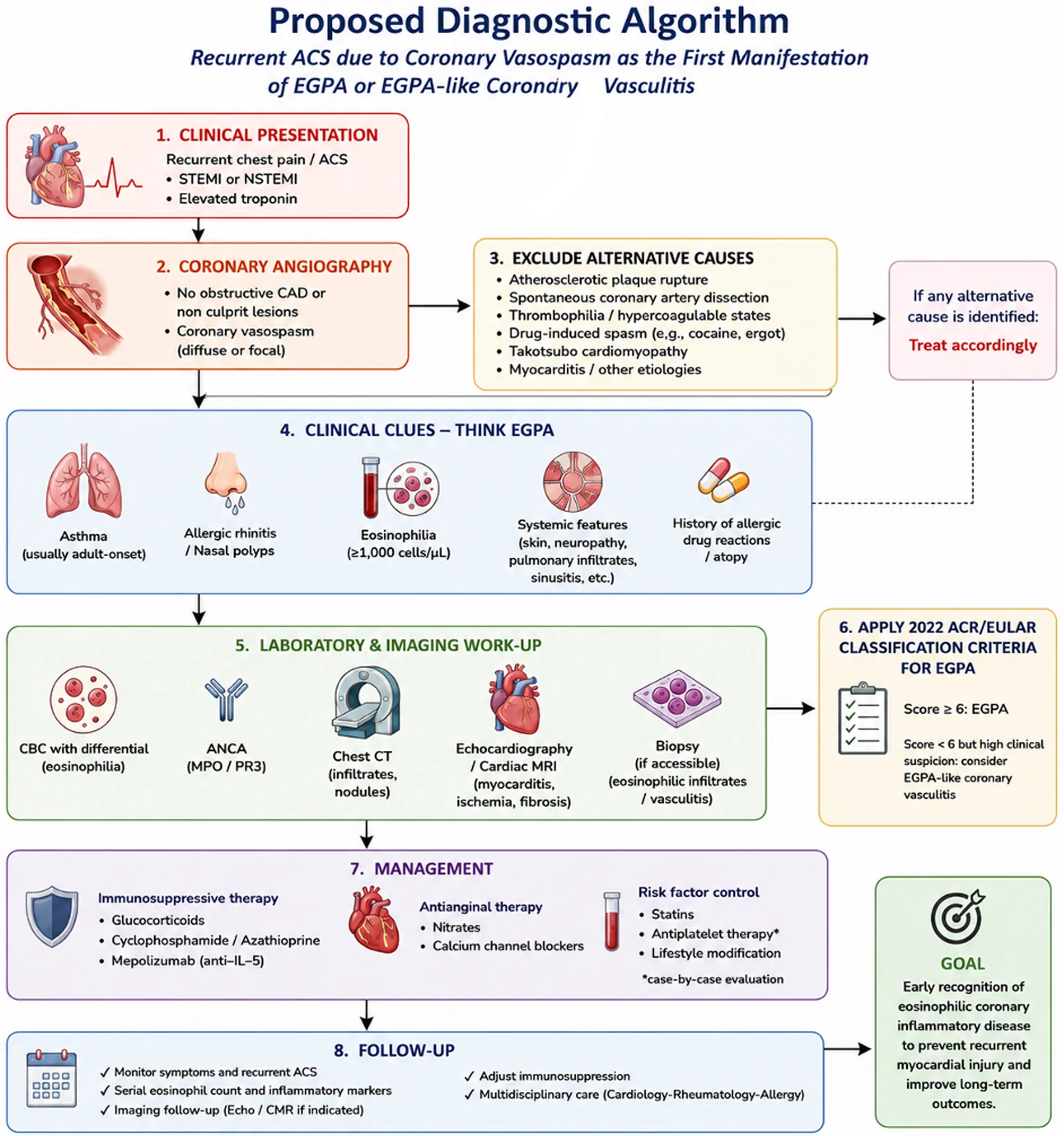
Proposed Diagnostic Algorithm for Recurrent ACS Associated With Eosinophilic Coronary Inflammatory Disease ACR/EULAR = American College of Rheumatology/European Alliance of Associations for Rheumatology; ANCA = anti-neutrophil cytoplasmic antibody; CAD = coronary artery disease; CBC = complete blood count; CMR = cardiac magnetic resonance imaging; CT = computed tomography; EGPA = eosinophilic granulomatosis with polyangiitis; IL = interleukin; MPO = myeloperoxidase; MRI = magnetic resonance imaging; NSTEMI = non-ST-segment elevation myocardial infarction; PR3 = proteinase 3; STEMI = ST-segment elevation myocardial infarction.

## Funding Support and Author Disclosures

The authors have reported that they have no relationships relevant to the contents of this paper to disclose.

**Take-Home Messages**

• Coronary vasospasm may be the presenting manifestation of EGPA.
• Recurrent ACS associated with asthma or eosinophilic disease suggests the presence of coronary vasculitis.
• Immunosuppressive therapy is crucial for long-term disease control.
